# Evaluation of the Risk of Spreading Endometrial Cell by Hysteroscopy: A Prospective Longitudinal Study

**DOI:** 10.1155/2009/397079

**Published:** 2009-05-25

**Authors:** Rievani de Sousa Damião, Reginaldo Guedes Coelho Lopes, Emilly Serapião dos Santos, Umberto Gazzi Lippi, Eduardo Borges da Fonseca

**Affiliations:** ^1^Departamento de Obstetrícia e Ginecologia, Hospital do Servidor Publico Estadual, “Francisco Morato de Oliveira”, Rua Pedro de Toledo 1800, 04039-901 São Paulo, SP, Brazil; ^2^School of Medicine, University of São Paulo, São Paulo, SP, Brazil

## Abstract

*Objective*. The aim was to assess the intraperitoneal spread of endometrial cells during hysteroscopy. *Study Design*. Seventy-six women were submitted to a hysteroscopy with CO_2_ under a low pressure. Group 1 had not previous diagnosis of endometrial cancer, and group 2 had previous diagnosis of endometrial cancer (stage I-92.3%). Two peritoneal washing samples were taken before (PW1) and immediately after (PW2) the procedure. The dissemination for the peritoneal cavity was defined by the presence of endometrial cells in the PW2; such cells should be absent in WP1. *Results*. Four patients were excluded for presenting endometrial cells in PW1. In the 72 patients left, there was no passage of cells for the peritoneal cavity. In group 1, 88% presented secretory endometrial phase with correlation of 80% between hysteroscopy and biopsy. *Conclusion*. Hysteroscopy performed under a low pressure of CO_2_ does not cause spreading of endometrial cells into the peritoneal cavity.

## 1. Introduction

Hysteroscopy has been established as the gold standard procedure to evaluate and to treat abnormal uterine bleeding [1, 2]. The uterine cavity can be thoroughly visualized and an endometrial biopsy specimen can be taken under hysteroscopic view [3, 4]. An endometrial carcinoma can be detected in 7%–10% of postmenopausal patients and 2%-3% of premenopausal patients submitted to hysteroscopy [5, 6]. In view of these, hysteroscopy is now considered as an important method in the investigation of endometrial cancer [3–6].

Hysteroscopy requires distention of the cavity with a gaseous or liquid medium at a pressure of 50–150 mmHg to allow complete visualization of the fundus and ostial areas. Liquid media used for this purpose include high-viscosity fluids such as 32% dextran 70 or low-viscosity fluids such as 5% dextrose, Ringer's or normal saline solution. The gas universally used for diagnostic hysteroscopy is carbon dioxide (CO_2_). There is evidence from observational studies that distension of uterine cavity could be associated with transtubal leakage of endometrial cells and tissue reflux into the peritoneal cavity [7–12] (Table 1).

It has also been demonstrated that liquid distention appears to have a higher leakage of endometrial cells compared to CO_2_ distention. On the other hand, there are studies, looking at CO_2_ distention, that presented contradictory results [7, 11, 13].

In fact, transtubal leakage of endometrial cell during hysteroscopy is of concern when investigating women complaining of abnormal uterine bleeding who are subsequently found to have endometrial malignancy. Several investigators have reported on retrograde seeding of endometrial carcinoma during hysteroscopy [14–16]. However, these results are controversial in view of different pressure and method of distention.

Although the clinical implication of such reflux has not yet been determined, in principle, it would be avoided in high-risk patients. The current evidence suggests that this would be best achieved with gaseous distention rather than with liquid distention. To investigate the influence of the uterine distention medium on tubal reflux, we conducted a prospective longitudinal study using hysteroscopy with CO_2_ at a pressure of 80 mmHg (low-pressure hysteroscopy) to assess the occurrence of eventual leakage of endometrial cells into the peritoneal cavity in women with and without endometrial cancer.

## 2. Material and Methods

Seventy-six patients were initially enrolled; sixty one underwent laparoscopy for tubal sterilization or other indications (group 1), and fifteen required laparotomy due to endometrial cancer (group 2).

The inclusion criteria were normal reproductive function with patent Fallopian tubes and no history of either tubal disease or tubal surgery, over 3 months of oral contraceptive use discontinuation, no history of pregnancy within the last year. The exclusion criteria were peritoneal cytology positive for endometrial cells after first peritoneal washing (PW1) and negative tubal patency test.

The study was carried out in a sequence of two stages. In the initial stage, either laparoscopy (in group 1) or laparotomy (in group 2) was performed, and peritoneal cells were collected for cytology study (control sample) by injecting 40 mL of normal saline solution (PW1) in the Douglas pouch, around the tubes and ovaries. When laparotomy was performed, a syringe containing 40 mL of saline solution was used for injection and aspiration of the peritoneal washing. When laparoscopy was performed, a second puncture was performed where a 5 mm Endopath trocar (Johnson & Johnson) was used for injection and aspiration of the peritoneal washing.

In the second stage, diagnostic hysteroscopy was performed by a standard hysteroscope with a 30° forward-oblique lens and 5 mm diagnostic sheath. An electronic Hamou hysteroflator (Karl Storz GmbH, Tuttingen, Germany), adjusted to a flow rate ≤50 mL/min and pressure ≤80 mmHg of CO_2_, was used to distend the intrauterine cavity. All hysteroscopies were performed by the same operator and lasted 4 minutes average. A second peritoneal washing (PW2) was performed using the same technique as that in stage one. Tubal patency was confirmed after the second sample was taken by transcervical injection of 20 mL methylene blue dye dilution through a cervical cannula. A selective endometrial sampling by hysteroscopy was performed immediately before PW2.

The two samples of the peritoneal washing (before hysteroscopy-PW1; after hysteroscopy-PW2) were fixed in 95% ethyl alcohol and centrifuged at 3000 g for 10 minutes. After being fixed by Papanicolaou staining technique, the samples were analyzed at 100× magnification. Cells were assessed morphologically. Endometrial and tubal cells were identified as nonciliated or ciliated epithelial cells, respectively. In addition, the samples were studied in a blind manner with respect to the diagnosis by an experienced cytopathologist. 

Positive peritoneal cytology was considered the primary endpoint of this study. Frequency distribution of ordered categorical variables was compared by means of exact Wilcoxon rank-sum test. Correlations between dichotomous variables were tested using Fisher's exact test. The data were analyzed using the chi-square test, and *P-*value of .05 was considered significant. The study was previously approved by the ethical committee.

## 3. Results

From the initial 76 patients, four (5.2%) were excluded due to positive peritoneal cytology after PW1. Two of these had endometrial cancer (stage IIIAG2), and two were in secretory phase of menstrual cycle. Therefore, 72 women participated, of which 13 had endometrial cancer (18.0%), were labeled group 2, and 59 who had no endometrial cancer (82.0%) were labeled group 1.

The characteristics of all these patients are presented in Tables 2  and 3. The previous diagnosis of endometrial cancer had been made by hysteroscopy plus biopsy. The interval time between diagnoses of cancer and surgery was 28 (24–40) days.

Among patients of group 2, 11 (84.6%) were in the postmenopausal phase and two (15.4%) in premenopausal phase. Hysteroscopy was indicated for abnormal vaginal bleeding in nine cases (69.2%) and for abnormal sonographic endometrial thickness in four cases (30.8%). The majority of these patients had been staged as I (92.3%).

In both groups, there were no endometrial cells in the second sample collected immediately after diagnostic hysteroscopy.

## 4. Comment

The data of this study demonstrate that diagnostic hysteroscopy performed under a low pressure of CO_2_ does not cause spreading of endometrial cells into the peritoneal cavity for both patients with and without early stage of endometrial cancer.

As hysteroscopy is largely indicated in patients with abnormal uterine bleeding, it becomes relevant to demonstrate whether this procedure is safe when underlying endometrial cancer is suspected. Abnormal endometrial cells reflux into the peritoneal cavity after diagnostic hysteroscopy which has been reported in about 16% of cases might increase the risk of recurrence [17, 18].

There is controversy regarding the potential dissemination of malignant endometrial cells into the peritoneal cavity through the Fallopian tubes during diagnostic hysteroscopy. However, retrospective studies have suggested that diagnostic hysteroscopy does not significantly increase the incidence of positive peritoneal cytology in patients with endometrial cancer [17, 19].

Stage and grade of endometrium cancer, intrauterine pressure, and the medium of distension used during the hysteroscopy are thought to be related with the spreading of malignant endometrial cells into the peritoneal cavity [2, 3, 5, 15, 17, 20, 21]. Nevertheless, there is no prospective study that could point at any of those factors as having a significant role in the spreading of malignant cells to the abdominal cavity.

Early recurrence of endometrial cancer within one year after surgical treatment has been reported as being caused by hysteroscopy dissemination of malignant cells [14–16]. The most important factor associated with transtubal spreading of endometrial cells during hysteroscopy procedure appears to be the intrauterine pressure used. Baker and Adamson [22] observed spreading of endometrial cell after diagnostic hysteroscopy using high intrauterine pressure, and Bettocchi et al. [23] have suggested that intrauterine pressure of 150 mmHg has a higher risk for cell dissemination. Leveque et al. [9] used intrauterine pressure of 150 mmHg and observed a positive peritoneal cytology in 37% of the cases. In contrast, positive peritoneal cytology is seen in about 1.0% when the intrauterine pressure was equal or below 100 mmHg [7, 8, 11, 12]. Baker and Adamson have demonstrated that no spread of endometrial cell occurs at intrauterine pressure equal or below 70 mmHg [22]. The main limitation of these studies was that the peritoneal cytology was not taken at the same time as hysteroscopy or as a previous cytology study before hysteroscopy.

Lo et al. [11] have also demonstrated that using a liquid medium for intrauterine distension has a higher association with positive peritoneal cytology after diagnostic hysteroscopy (14% versus 1.4%). Hence, the risk of spreading cell into the peritoneal cavity is lower when this was done by gaseous medium under a low pressure to distend the uterine cavity [3, 10, 14, 16, 24].

In our study, we performed diagnostic hysteroscopy using intrauterine pressure no greater than 80 mmHg and CO_2_ gas to distend the intrauterine cavity. Also, peritoneal cytology was performed before as well as after hysteroscopy. All included patients in the study had absent endometrial cells in the first washing. None of our cases showed positive peritoneal cytology after hysteroscopy.

In conclusion, diagnostic hysteroscopy using intrauterine pressure no greater than 80 mmHg and CO_2_ gas to distend the intrauterine cavity appears to be a safe procedure in high-risk patient for endometrial cancer. However, further studies are required to assess endometrial cell spreading after diagnostic hysteroscopy in different stages of endometrial cancer with long followup.

## Figures and Tables

**Table 1:** Studies reporting on the association between hysteroscopy and positive peritoneal cytology where *N*: number of cases; PW: peritoneal washing.

Author	*N*	Indication for surgery	Hysteroscopy		Laparoscopy
			Distention	Pressure	Positive PW
Ranta et al. [7]	51	Infertility	CO_2_	80 mmHg	8/51 (15.6%)
Sagawa et al. [8]	24	Endometrial cancer	Glucose solution or dextran	50 cmH_2_O	2/24 (8.4%)
Leveque et al. [9]	19	Endometrial cancer	CO_2_ NaCl 0.9% (2 cases)	150 mmHg	7/19 (36.8%)
Gücer et al. [10]	31	Endometrial cancer	NaCl 0.9%	200 mmHg	3/31 (9.7%)
Lo et al. [11]	70	Endometrial cancer	CO_2_	100 mmHg	1/70 (1.4%)
Lo et al. [11]	50	Endometrial cancer	NaCl 0.9%	100 cmH_2_O	7/50 (14%)
Solima et al. [12]	40	Endometrial cancer (Stage I or II)	NaCl 0.9%	40 mmHg	2/40 (5%)

**Table 2:** Characteristics of patients with benign endometrial cytology (group 1).

	*N*	%
Numbers of patients	59/72	81.9
Age years (range)	35 (17–41)	—
Nulliparous	10	17.0
Laparoscopy indication		
* *Tubal sterilization	36	61.0
* *Hysterectomy	14	23.7
* *Ovarian mass	6	10.2
* *Chronic pelvic pain	3	5.1
Phase of menstrual cycle		
* *Secretory	52	88.1
* *Proliferative	7	11.9

**Table 3:** Characteristics of patients with cancer (group 2); group1: <5% of a nonsquamous or nonmorular solid growth pattern; group2: 6–50% of a nonsquamous or nonmorular solid growth pattern.

	*N*	%
Number of patients	13/72	18.1
Age years (range)	57 (51–79)	—
Hysteroscopy indication		
* * Abnormal uterine bleeding	9	69.2
* * Abnormal thickness	4	30.8
Endometrial		
Stages*		
* *IA1	5	38.4
* * IB2	3	23.1
* * IC1	3	23.1
* * IC2	1	7.7
* * IIIIC2	1	7.7

Corpus Cancer Staging according to FIGO Stages—1988 Revision.
